# Low–Threshold and High Intensity Random Lasing Enhanced by MnCl_2_

**DOI:** 10.3390/ma9090725

**Published:** 2016-08-24

**Authors:** Zhenzhen Shang, Mingchao Yang, Luogen Deng

**Affiliations:** School of Physics, Beijing Institute of Technology, Beijing 100081, China; enenshang.2016@gmail.com (Z.S.); yangmingchao168@gmail.com (M.Y.)

**Keywords:** MnCl_2_, random lasing, polymer dispersed liquid crystal, low-threshold, 42.60.Fc

## Abstract

Energy transfer is known to have a significant influence on random lasers. However, the study about the effect of energy transfer between metallic salt and dye molecules on random lasers is still lacking at present. Here, we investigate random lasing actions in Pyrromethene-597 (PM597), PM597-doped MnCl_2_ (manganese (II) chloride), PM597-doped polymer-dispersed liquid crystal (PDLC) and PM597-doped PDLC with MnCl_2_ capillary systems. We find that random lasing of the systems with MnCl_2_ exhibits lower threshold, higher intensity, sharper peak and variable resonance wavelength in comparison with the systems without MnCl_2_. This behavior is closely related to the decrease of fluorescence quenching effect and the enhancement of local field induced by energy transfer between MnCl_2_ and PM597. Red-shift of wavelength is observed with increasing dosage concentration of MnCl_2_ in the PM597-doped PDLC with MnCl_2_ system. Through the analysis of single-shot emission spectra of PM597-doped PDLC without and with MnCl_2_ systems, the role of MnCl_2_ in the coupling of lasing modes is confirmed. Lengths of laser oscillation cavities of the PM597-doped PDLC without and with MnCl_2_ systems are calculated by a power Fourier transform (PFT) analysis of their emission spectra. It well accounts for the effect of MnCl_2_ on the variation of the oscillation cavity.

## 1. Introduction

Random lasers have attracted widespread attention from scientists due to its potential applications, such as miniature spectroscopy, speckle-free projection, large area holographic laser displays and medical diagnostics [1,2,3,4]. Conventional lasers are built on well defined cavities and have fixed emission wavelength, fixed lasing mode as well as good direction. In contrast, random lasers are mirrorless, where a large number of modes with uncorrelated phases can be excited simultaneously. This leads to an emission with low threshold and low spatial coherence but high spectral intensity. Spatial modes in a random laser are dominated by the resonant frequency of gain-scattering system, which are inhomogeneous and irregular [3,4,5]. Light scatterers and optical gain material are usually randomly embedded in a host medium, so random lasing is usually explained by multiple light scattering, which causes spatially distributed feedback of light and results in random lasing [6,7,8,9]. However, in systems where gain material and scatterers are separated, the random lasing mainly attributes to spatially localized feedback of light [10,11]. In general, two types of feedback mechanisms are identified in random lasers, resonant (field feedback) and non-resonant (intensity feedback). If resonant feedback is predominately involved, emission spectrum will show sharp spikes. Nevertheless, emission spectrum merely exhibits narrowing phenomenon when random laser is triggered by non-resonant feedback [12]. Therefore, scatterer material and optical gain material are two vital elements in random lasing. To date, random lasers have been widely studied in various dielectric materials systems, such as semiconductor powder [13], liquid crystals [14,15], polymer materials [16], human tissues [17], bone tissues [6] and fibers [18].

Speckle-free imaging using laser illumination was reported, which is based on the low spatial coherence of random lasing [19]. Leonetti et al. engineered a mode-selective pumping of a random laser formed by a self-assembled cluster of nanoparticles and realized the mode-locking transition of radom laser [20]. A novel dopamine sensing and measurement technique controlled by aggregation of gold nanoparticles in random laser was demonstrated [21]. However, the tunability of random lasers has caused more concern of the researchers. They have utilized a variety of methods to control the random lasers, including adjusting the concentration of scatterers and dye [22], using external electric field [23] and applying the enhancement effect of localized surface plasmon [24,25]. Recently, a high-efficient random laser which consists of a freestanding polymer membrane embedded with silver nanoparticles was demonstrated, where the plasmon-assist waveguide resonance lowered the threshold of the random laser [26]. A polarization-modulated random fiber laser was experimentally verified for the first time [27]. Lee et al. designed an electrically and thermally controllable nanoparticle random laser in a well-aligned dye-doped liquid crystal cell [28]. Tunable dye random lasers supported by energy transfer were demonstrated to be an important tool in a wide variety of fields [29,30].

Nevertheless, achieving simultaneously tunability and flexibility of random lasers by applying low-cost and good performance materials is still a challenge for researchers at present. In this manuscript, we investigate random lasers with coherent feedback in dye-doped MnCl_2_ (manganese (II) chloride) glass capillaries for the first time. By comparing random lasing in systems without and with MnCl_2_, we note that the random lasing from the latter system exhibits lower threshold, higher intensity and sharper peak. Through the analysis of single-shot emission spectra and cavity lengths of PM597-doped polymer-dispersed liquid crystal (PDLC) without and with MnCl_2_ systems, the effect of energy transfer caused by MnCl_2_ on random lasers is verified. The results reported here offer a simple and straightforward method for the low-cost, small size and flexible shape fabrication of random laser devices.

## 2. Experiments

**Figure 1:**
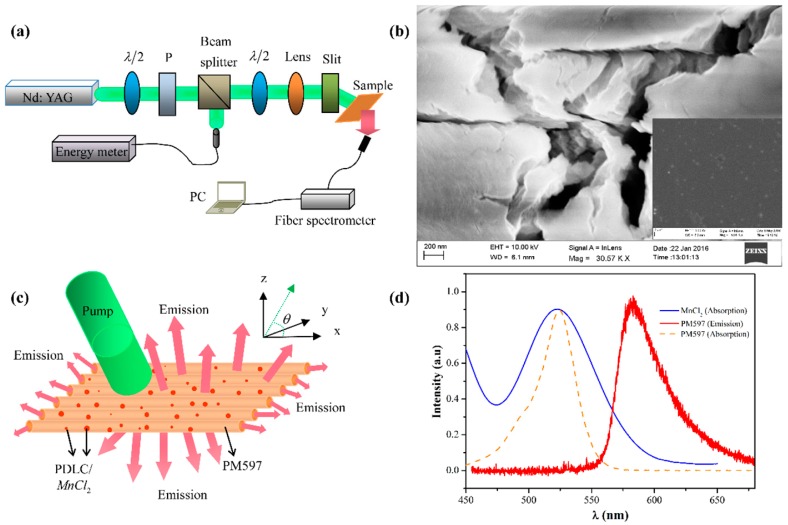
(**a**) Experimental setup for measuring random lasing spectra; (**b**) SEM image of solid MnCl_2_·4H_2_O. Inset: SEM image of water-solution of MnCl_2_, where spots with average diameter 250 nm are MnCl_2_; (**c**) Schematic diagram of lasing emission in the sample; (**d**) Red line is spontaneous emission spectrum of PM597, blue line is absorption spectra of MnCl_2_ and dotted line is absorption spectra of PM597.

Table 1 indicates the ingredients of samples in these five systems. The optical gain material in our samples is a liquid solution of Pyrromethene-597 (PM597) dispersed in ethanol. MnCl_2_ used in the samples is a water solution containing MnCl_2_·4H_2_O (6.187 g, *n* = 1.985) and deionized water (2 mL), which is heated on sample heater with 80 °C for 15 min. By mixing water-MnCl_2_ (0.2 mL) with PM597 (1 mg) in ethanol (0.1 mL), PM597-doped MnCl_2_ solution was obtained after stirring for 30 min. The polymer-dispersed liquid crystal (PDLC) was prepared by mixing the following component. Trimethylolpropane triacrylate monomer (42.06 wt %), cross-linking monomer, N-vinylpyrrolidone, (8.29 wt %), photoinitiator, rose Bengal (0.58 wt %), coinitiator, N-phenylglycine (1.53 wt %), surfactant, octanoic acid (8.82 wt %), nematic liquid crystal, E7 (37.32 wt %, *n_o_* = 1.5216, *n_e_* = 17462). PM597-doped PDLC solution was prepared by dissolving PM597 (1 mg) in ethanol (0.1 mL) mixing with PDLC (0.2 mL) followed by stirring for half an hour. By further adding water-MnCl_2_ in the above PM597-doped PDLC solution, PM597-doped PDLC with MnCl_2_ solution was obtained. Two kinds of PM597-doped PDLC with MnCl_2_ solutions containing deionized water-MnCl_2_ 0.1 and 0.2 mL were prepared, respectively. All the prepared solutions were shaken for 40 min by ultrasonic. Mixed solutions were poured into glass capillaries by capillary action. Capillaries used in the experiment are 50 cm long, whose diameter is 0.5 mm. The sample illustration is shown in Figure 1c. SEM images of solid MnCl_2_·4H_2_O and water-solution of MnCl_2_ are shown in Figure 1b, in which the inset depicts distribution and size of MnCl_2_ in the water solution. Figure 1d plots the absorption spectra of MnCl_2_ and dyes PM597, as well as the spontaneous emission spectrum of PM597.

Experimental setup is shown schematically in Figure 1a, where samples are pumped with the second harmonic of a mode-locked Nd:YAG laser (λ = 532 nm, 10 Hz repetition rare, 8 ns pulse duration). A half-wave plate (λ/2 for 532 nm), a polarizer (P) and a neutral beam splitter are used in order to vary the incident pulse energy. The incident pulse is divided into two sub-beams by beam splitter, one is collected as a reference beam by energy meter and the other is used as pump light. The pump light is converged by an optical lens and form a strip pump light with the length 9 mm and the variable width 0.06 to 0.16 mm through an adjustable slit. Random lasing emission (see Figure 1c) of samples is collected by a spectrometer connected to a computer from the outside surface and the both ends of the capillaries.

## 3. Results and Discussion

**Figure 2:**
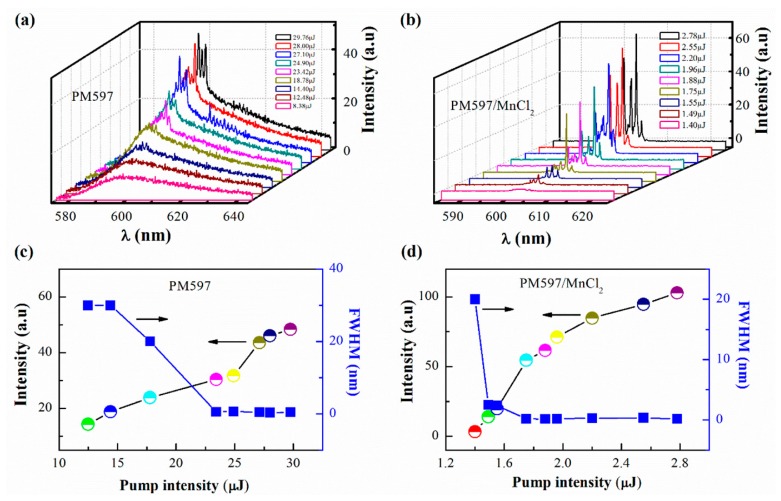
Emission spectra as a function of pump energies for (**a**) PM597 and (**b**) PM597-doped MnCl_2_ systems, respectively. Main-peak intensities and FWHM of emission spectra versus pump energies for (**c**) PM597 and (**d**) PM597-doped MnCl_2_ systems.

Figure 2a depicts the dependence of emission spectra on pump energy for PM597. Below threshold, the emission spectrum is dominated by a broad band centered at λ ~ 598 nm with a full width at half maximum (FWHM) of 30 nm, indicating the spontaneous emission of PM597. This band exhibits an obvious red shift of about 20 nm with respect to the spontaneous emission of PM597 shown in Figure 1d, which stems from the self-absorption of PM597 emission [31]. A sharp peak with FWHM of 0.47 nm suddenly emerges at the pump energy of 23.42 μJ, which is enhanced with the increasement of pump energy. The sharp peak may be owing to the spatially localized feedback caused by the optical gain material and the internal roughness of capillary tubes. Amplified spontaneous emission (ASE) from the pumped area reaches the internal rough wall of capillary tubes after being poorly attenuated in the unpumped dye and is back-scattered. The back-scattered light returns to the gain material and closes an optical feedback loop, motivating the lasing action [10,11]. In addition, spatially distributed feedback that originates from grains of solid PM597 not fully dissolved in the capillary tubes may also be responsible for this phenomenon. The random distribution of the grains provides multiple scattering when ASE is running in the capillary tubes and results in random lasing. As shown in Figure 2b, as MnCl_2_ is doped in the PM597, more modes appear in the spectrum. Moreover, the number and the spectral positions of the modes remain constant over time and upon pump energies. This suggests that PM597-doped MnCl_2_ system is more stable than the PM597 system. Just like Figure 2a, only the spontaneous emission curve of PM597 arises at low pump energy. When the pumping energy exceeds 1.75 μJ, a set of sharper peaks centered at λ ~ 599 to 604 nm with FWHM of 0.14~0.19 nm emerge on the top of the spontaneous emission band, which are sharper than that in PM597 system. Emission intensity and FWHM versus pump energies for both PM597 and PM597-doped MnCl_2_ systems are plotted in Figure 2c,d, where the latter system shows lower threshold and higher intensity. In the PM597 system, higher pump energy is required to achieve lasing because the dye molecules are directly excited by pump and its concentration is too low. In the PM597-doped MnCl_2_ system, the specific random distributions of the grains of solid PM597 not fully dissolved in the tubes are changed as the doping of MnCl_2_, which influence the spatial distribution of modes and consequently the spectrum [10,11]. Additionally, a large overlap of absorption spectra of MnCl_2_ and PM597 can be found in Figure 1d and there is partial overlap between the absorption spectrum of MnCl_2_ and the spontaneous emission spectrum of PM597. This implies that energy transfer may exist between MnCl_2_ and PM597, where PM597 dye molecule emits a photon which is subsequently absorbed by MnCl_2_ molecule, on the other hand, the excited MnCl_2_ molecule may transfer its excitation energy to a ground-state of PM597 dye [29,30,32]. Thus, except the effect of spatially localized feedback and spatially distributed feedback mentioned in Figure 2a, energy transfer between MnCl_2_ and PM597 may play a more important role for these unique characteristics of emission spectra in the PM597-doped MnCl_2_ system. It lowers the fluorescence quenching effect and enhances the local field of random microcavity. The enhancement of local field of random microcavity shifts the lasing wavelength of PM597 and boosts the lasing. The decrease of the fluorescence quenching effect extends the spontaneous emission lifetime τ of dye molecules. As we know, threshold population inversion of random gain system can be expressed as follows [31,33]

(1) $$n_{th}=4\pi c\tau n^{2}(2\alpha l-\ln R)/l\Omega (\lambda )$$

Here, α is the absorption coefficient at a laser wavelength, *l* is the dimension of active laser medium, *R* represents the effective reflection coefficient, *n* stands for the index of refraction of the gain material, Ω(λ) is the parameter related to emission spectrum and *c* is the velocity of light in vacuum. Hence, in the regime, the longer the spontaneous emission lifetime is, the quicker the threshold condition will be achieved. That is, the extended spontaneous emission lifetime accelerates the threshold population inversion of the random gain system and lowers its threshold.

**Figure 3:**
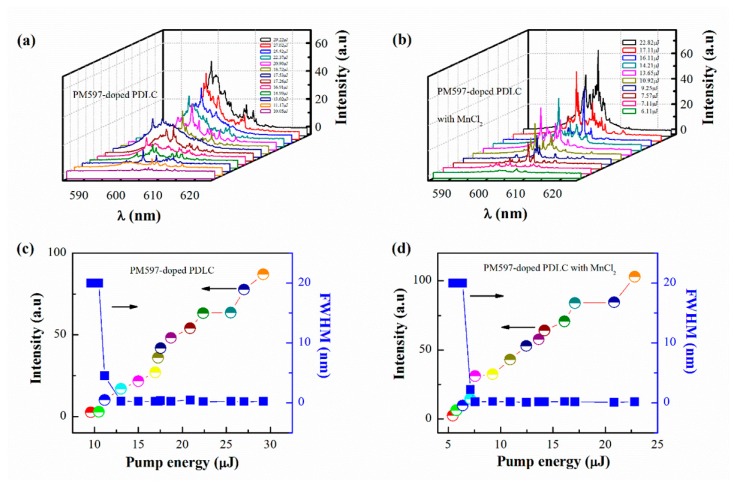
Emission spectra as a function of pump energies for (**a**) PM597-doped PDLC and (**b**) PM597-doped PDLC and MnCl_2_ (0.1 mL) systems, respectively. Main-peak intensities and FWHM of emission spectra versus pump energies for (**c**) PM597-PDLC and (**d**) PM597-doped PDLC and MnCl_2_ (0.1 mL) systems, respectively.

**Figure 4:**
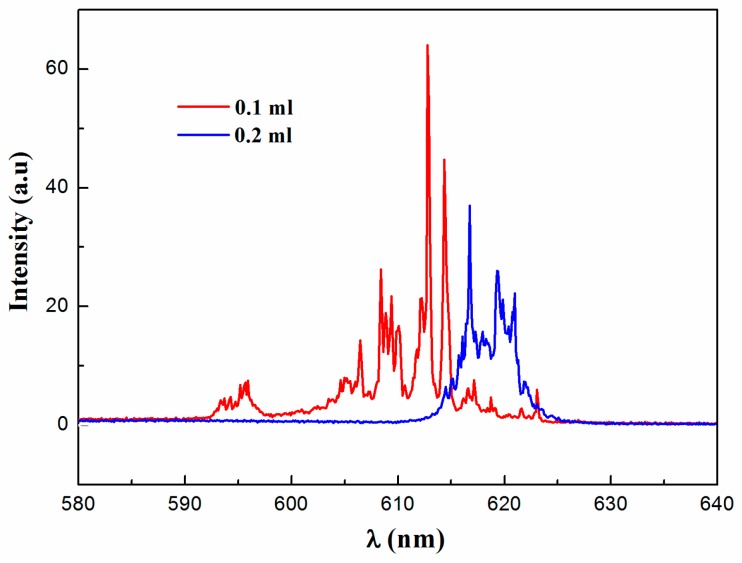
Emission spectra at 0.1 and 0.2 mL MnCl_2_ dosage concentration in PM597-doped PDLC with MnCl_2_ system at same pump energy 7.65 μJ.

To further confirm the effect of MnCl_2_ on random lasing, random lasing in PM597-doped PDLC with and without MnCl_2_ systems is investigated. Figure 3a describes the evolution of emission spectra against pump energy for PM597-doped PDLC system, where it clearly indicates a threshold at 13.02 μJ. A spontaneous emission band with FWHM of 20 nm emerges at low pump energy. A narrow peak located at λ ~ 606 nm with FWHM of 0.45 nm arises upon the threshold. Above the threshold, more and more sharp peaks (with FWHM of 0.23 nm around) appear on the broad emission spectra. These sharp peaks are mainly related to multiple light scattering caused by the birefringence of the anisotropic liquid crystals. The multiple scattering lengthens the dwell time of photons in the gain system and leads to amplification [14]. However, the doping of MnCl_2_ (0.1 mL) in the PM597-doped PDLC system results in distinct emission profiles, as presented in Figure 3b. For instance, the system with MnCl_2_ exhibits more discrete sharp laser spikes and narrower emission peaks with FWHM of 0.15 nm. As illustrated in Figure 3c,d, lower threshold (7.57 μJ) and higher intensity can also be observed in the PM597-doped PDLC with MnCl_2_ (0.1 mL) system compared with the PM597-doped PDLC system. The observations of the distinct random lasing in PM597-doped PDLC with MnCl_2_ are associated with two aspects. On the one hand, some molecule clusters containing PDLC and MnCl_2_ may be formed after adding MnCl_2_ in the PM597-doped PDLC system. Furthermore, the alignment of liquid crystals molecules is varied when MnCl_2_ is doped in the PM597-doped PDLC system. The random distributions of the molecule clusters and the liquid crystals enhance the multiple light scattering and the localization of the random system, which cause different repulsion and coupling interaction among lasing modes. On the other hand, the transfer energy between PM597 and MnCl_2_ varies the fluorescence quenching effect and the local field of random microcavity. Figure 4 plots the emission spectra for different MnCl_2_ dosage concentration in the PM597-doped PDLC with MnCl_2_ system at same pump energy (7.65 μJ). This emission is shifted to long wavelength side of the spectrum due to the re-absorption of dye molecules as the doping density of MnCl_2_ increases from 0.1 to 0.2 mL [34]. In addition, the efficiency of energy transfer between MnCl_2_ and dye molecule related to MnCl_2_ dosage concentration will also affect the position of the emission.

**Figure 5:**
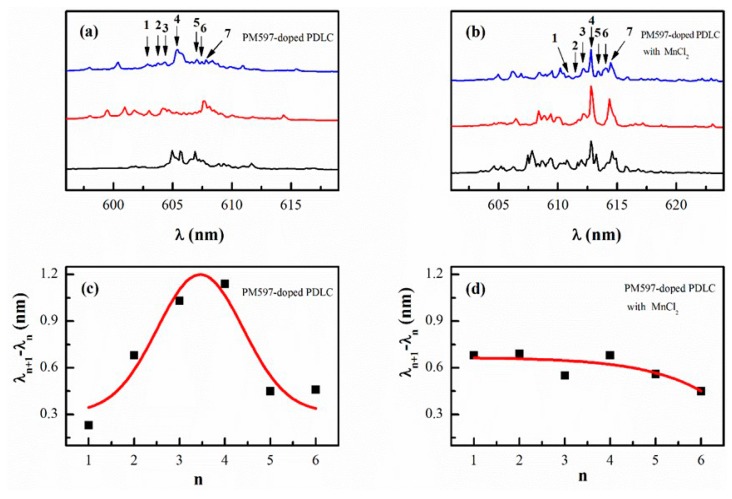
Single-shot emission spectra at different time with same pumped position, same pumped area (*A* ~ 0.54 mm^2^) and same pump energy for (**a**) PM597-doped PDLC (14.25 μJ) and (**b**) PM597-doped PDLC and 0.1 mL MnCl_2_ (7.65 μJ), respectively; (**c**,**d**) Mode separation of adjacent modes versus the lasing mode derived from (**a**,**b**), respectively.

To quantitatively analyze our experimental findings, single-shot emission spectra recorded at same pumped position and different time with pumped area *A* ~ 0.54 mm^2^ for PM597-doped PDLC without and with MnCl_2_ (0.1 mL) systems are presented in Figure 5a,b, in which pump energies are 14.25 and 7.65 μJ, respectively. In principle, the nature of a random gain-scattering system largely depends on the variance of configuration of scattering centers. For a dynamic system, particles in it will move randomly and cause variation of oscillation cavities, which will change lasing frequency of emission spectrum [24,25]. However, particles in static system will keep immobile basically, so it will not change the lasing frequency. We note that the emission spectra of PM597-doped PDLC with MnCl_2_ do not exhibit strongly chaotic behavior, while that of PM597-doped PDLC show chaotic behavior (see Figure 5a,b). Hence, oscillation cavities of random lasers are fixed in the former system and are variable in the latter system. To quantitatively confirm this fact, we choose several lasing modes around the central lasing frequency as shown in Figure 5a,b. Corresponding mode separations between adjacent modes are demonstrated in Figure 5c,d. The even separations of lasing modes are observed in PM597-doped PDLC with MnCl_2_ system, suggesting strong spatial confinement of lasing modes caused by MnCl_2_. However, intense fluctuation of the separations in PM597-doped PDLC system implies strong competition between lasing modes [35]. Consequently, MnCl_2_ decreases the number of coupling of lasing modes in the random system and makes the system more stable.

**Figure 6:**
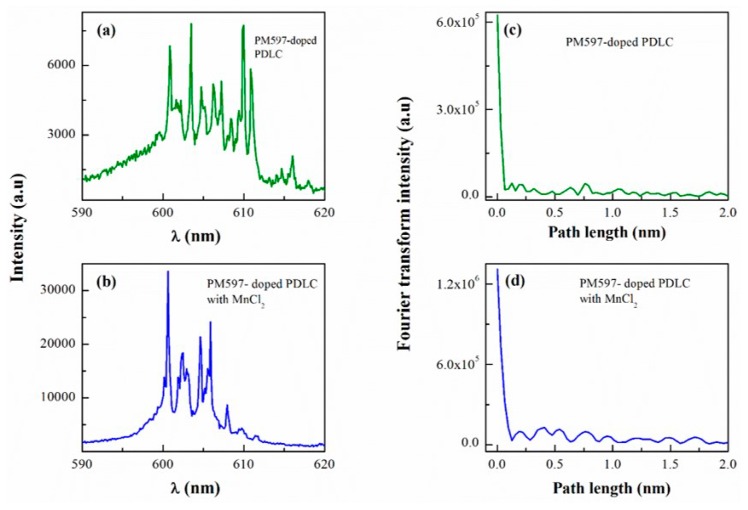
Emission spectra of (**a**) PM597-doped PDLC and (**b**) PM597-doped PDLC and MnCl_2_ (0.1 mL) systems with pump energy 14.75 μJ and pumped area *A* ~ 0.06 mm^2^; (**c**,**d**) The power Fourier transform (PFT) of corresponding emission spectra in Figure 6a,b.

To gain further insight into the variation of oscillation cavity length induced by MnCl_2_, we performed a power Fourier transform (PFT) analysis for single-shot emission spectra of PM597-doped PDLC without and with MnCl_2_ (0.1 mL) systems, respectively. Figure 6a,b present single-shot emission spectra with same pumped area (*A* ~ 0.06 mm^2^) and pump energy (14.75 μJ) for both systems, the corresponding PFT of the two spectra are plotted in Figure 6c,d. As we know, positions of the peaks in PFT spectrum are given by [17,34,36]

(2) $$d_{m}=nLm/\pi$$

where *m* is an integer denoting the Fourier transform harmonics, *L* corresponds to oscillation cavity length and *n* represents the refraction index of gain system. Thus, oscillation cavity length will be given as follows

(3) $$L=\pi d_{1}/n$$

where *d*_1_ is the first peak position of PFT spectrum. Based on the values of *d*_1_ (see Figure 6c,d) and *n* (*n* = 1.7462 and 1.889 for PM597-doped PDLC without and with MnCl_2_), Equation (3) gives the *L* values of ~228 and 316 μm, corresponding to the former and the latter system, respectively. The longer cavity length implies extended lifetime of photons in the system, which is consistent with the variation of the fluorescence quenching effect related to energy transfer mentioned above. In the future, we will apply challenging fluorescence lifetime measurement and scanning near-field optical spectroscopy to understand the nature of the laser microcavity and try to fabricate some simple optical devices based on random lasers.

## 4. Conclusions

In this work, we have reported a low-threshold and high intensity coherent random laser in gain systems with MnCl_2_. The overlap in three lines shown in Figure 1d is a key factor for the unique characteristics of the coherent random laser, which induces an energy transfer to boost the random lasing. The analyses of single-shot emission spectra from PM597-doped PDLC without and with MnCl_2_ systems confirm the effect of MnCl_2_ on the stability of the systems. In addition, red-shift of sharp peaks is observed with increasing the dosage concentration of MnCl_2_ in the PM597-doped PDLC with MnCl_2_ system. Cavity lengths of the PM597-doped PDLC without and with MnCl_2_ systems are calculated by a Power Fourier Transform analysis of emission spectra, which directly provides an evidence for the extended lifetime of photons in random system caused by doping MnCl_2_. This work demonstrates a simple and straight method of controlling threshold, intensity and wavelength of random laser by using MnCl_2_ for the first time. We believe MnCl_2_, as a kind of low-cost material with excellent random laser performance in random gain systems, is very suitable for the manufacture of optical devices based on random lasers in the future.

## Figures and Tables

**Table 1:** The ingredients of samples in five systems.

	Sample	PM597	PM597-Doped MnCl_2_	PM597-Doped PDLC	PM597-Doped PDLC with MnCl_2_	PM597-Doped PDLC with MnCl_2_
Ingredient						
PM597 (mg)		1	1	1	1	1
Ethanol (mL)		0.1	0.1	0.1	0.1	0.1
PDLC (mL)		-	-	0.2	0.2	0.2
Water-MnCl_2_ (mL)		-	0.2	-	0.1	0.2
